# Measuring Outdoor Walking Capacities Using Global Positioning System in People with Multiple Sclerosis: Clinical and Methodological Insights from an Exploratory Study

**DOI:** 10.3390/s21093189

**Published:** 2021-05-04

**Authors:** Coralie Delahaye, Dorine Chaves, Florian Congnard, Bénédicte Noury-Desvaux, Pierre-Yves de Müllenheim, on behalf of the SOCOS Group

**Affiliations:** Institute of Physical Education and Sports Sciences (IFEPSA), West Catholic University (UCO), F-49136 Les Ponts-de-Cé, France; delahaye.coralie@hotmail.fr (C.D.); chaves.dorine97@gmail.com (D.C.); fcongna2@uco.fr (F.C.); bnoury@uco.fr (B.N.-D.)

**Keywords:** functional capacity, overground walking, ambulatory assessment, wearable sensor

## Abstract

We aimed at showing how Global Positioning System (GPS) along with a previously validated speed processing methodology could be used to measure outdoor walking capacities in people with multiple sclerosis (MS). We also deal with methodological issues that may occur when conducting such measurements, and explore to what extent GPS-measured outdoor walking capacities (maximal walking distance [MWD_GPS_] and usual walking speed) could be related to traditional functional outcomes (6-min total walking distance) in people with MS. Eighteen people with MS, with an Expanded Disability Status Scale score ≤6, completed a 6-min walking test and an outdoor walking session (60 min maximum) at usual pace during which participants were wearing a DG100 GPS receiver and could perform several walking bouts. Among the 12 participants with valid data (i.e., who correctly completed the outdoor session with no spurious GPS signals that could prevent the detection of the occurrence of a walking/stopping bout), the median (90% confidence interval, CI) outdoor walking speed was 2.52 km/h (2.17; 2.93). Ten participants (83% (56; 97)) had ≥1 stop during the session. Among these participants, the median of MWD_GPS_ was 410 m (226; 1350), and 40% (15; 70) did not reach their MWD_GPS_ during the first walking bout. Spearman correlations of MWD_GPS_ and walking speed with 6-min total walking distance were, respectively, 0.19 (−0.41; 0.95) and 0.66 (0.30; 1.00). Further work is required to provide guidance about GPS assessment in people with MS.

## 1. Introduction

Multiple sclerosis (MS) is a chronic inflammatory disease of the central nervous system that affects more than two million people worldwide [1]. MS can cause various neurological and functional disorders, such as alterations in sensation, vision, cognition, balance, and walking capacities [2]. Among the functional limitations encountered by people with MS, those concerning walking are of particular importance due to their negative impact on physical activity and quality of life [3].

Assessing walking capacities, both with walking distance metrics (e.g., total distance during a six-minute walking test) and walking speed metrics (e.g., usual walking pace), has clinical interests in people with MS, such as understanding to what extent mobility may be altered during daily life, and quantifying functional benefits from therapies that would aim at improving mobility [4,5]. Although self-perception of walking capacities in people with MS is clinically relevant information, objective measurements are needed to have a more accurate picture of the capacities of the patient, especially since subjective measurements using scales may not reflect the results obtained from objective measurements [6].

Several laboratory-based walking tests have been used to objectively measure walking capacities of people with MS [6]. However, these tests have some drawbacks, including the need of an appropriate space to conduct the test, and the fact that capacities in real-life contexts may not be well reflected by laboratory-based measurements [6]. In contrast, outdoor evaluations, that can be implemented in large areas and for a relatively long duration, may allow us to better reproduce and more directly measure the daily life outdoor walking capacities of patients in terms of walking speed, endurance, and pattern (e.g., inter-walking bout variability in walking speed, variability in recovery duration, etc. [7]), with the possibility to perform the evaluations close to home [8] and thus with better accessibility for the patients with sufficient functional capabilities. Thus, some studies [9,10] have proposed an evaluation of walking capacities of people with MS in natural (i.e., outdoor) contexts where walking capacities were characterized using the greatest distance, measured by Global Positioning System (GPS), that a patient could walk before stopping due to symptoms (e.g., fatigue), also called “maximal walking distance”. Unfortunately, some concerns could be raised from these studies regarding the validity of the GPS-measured maximal walking distances in view of the implemented methodologies. Indeed, in the study by Créange et al. [9], the patients were asked to perform a single maximal walking bout (i.e., the greatest distance as possible) at usual pace around a hospital. However, it is unknown as to whether allowing several walking bouts to be performed, rather than a single one as in the study by Créange et al. [9], would allow the patients to reach a higher maximal walking distance, as it has been observed in other diseases inducing walking limitations [11]. In the study by Dalla-Costa et al. [10], the result used to characterize patients’ walking capacity was the mean of the daily greatest walking distances measured during several days in real-life contexts. However, in this last study, there was no information about how GPS data were processed to detect walking bouts and then calculate walking distance during daily life. Moreover, some studies conducted in other populations with walking limitations [7,8,12] suggest that, in addition to maximal walking distance, other outcomes could be of interest when measuring outdoor walking capacities in people with MS, such as outdoor usual walking speed, which is a parameter that may be needed to reveal the improvement of the functional status following a treatment procedure such as surgery [12].

While GPS allows easy outdoor measurements of speed and distance, one of the methodological challenges raised when analyzing data from an evaluation that includes multiple walking bouts is to correctly discriminate the actual walking bouts from the actual stopping bouts for then calculating walking distance. Previous works [13,14] have shown that a semi-automatic speed processing methodology can correctly detect walking bouts and accurately estimate walking speed and distance. While this procedure has been successfully implemented in patients with intermittent claudication [7,8,11], it is unknown as to whether it is usable in MS patients with various functional profiles, as reflected for example by various usual walking speeds or capacities of maintaining balance and pace on nonlinear paths. Thus, the objective of the present study was three-fold:

- illustrate, using fully open materials, how GPS data, in particular speed, obtained during an outdoor evaluation allowing multiple walking bouts, could be used along with a previously validated speed processing methodology [13,14] to characterize walking capacities in people with MS;
- highlight methodological issues that may occur when implementing an outdoor walking evaluation with GPS measurements in people with MS; and
- explore the construct validity of outdoor maximal walking distance and outdoor usual walking speed as functional outcomes in people with MS.

## 2. Materials and Methods

### 2.1. Recruitment and Framework of the Study

We recruited a convenience sample of patients with MS in three different structures: a rehabilitation clinic in Angers, a sports association in Nantes, and another sports association in Saint-Nazaire. In Angers, after having received medical clearance from a physical and rehabilitation physician of the clinic, patients followed a program that included three exercise sessions per week. The program aimed at increasing physical condition (aerobic fitness, muscular fitness, flexibility, and balance). In Nantes and Saint-Nazaire, patients were referred to the sports association by physicians working at the hospital centers of Nantes and Saint-Nazaire, respectively. In the two sports associations, patients had the possibility to participate in various activities including light-to-moderate aerobic physical activities and resistance exercises. In Nantes and Saint-Nazaire, patients participated in the physical activities programmed by the sport association one to two times a week.

To participate in the present study, patients with MS had to be ≥18 years old, be insured under the French social security system (according to French law), have received medical clearance for participating in light-to-moderate walking activities, and give written informed consent to participate in the study. Patients could not be included in the study when they could not understand the description of the study, when MS was not the main cause of their exercise limitations, and when their medical treatment was modified during the last three months. Eighteen eligible patients volunteered to participate: 6 in Angers, 4 in Nantes, and 8 in Saint-Nazaire.

For each participant, the study consisted of completing the following: (i) an interview to obtain clinical and functional information; (ii) a 6-min walking test; and (iii) an outdoor walking session with GPS measurements. The walking test and the outdoor walking session were parts of the program or the activities that the structure routinely implemented under the supervision of adapted physical activity specialists. We conducted the present study in accordance with the principles of the Declaration of Helsinki and with the approval from our institutional review board and from the staffs responsible for the management of the patients.

### 2.2. Protocol

We collected the physical characteristics of the participants (sex, age, height, body mass) either from their respective medical records for the patients from Angers or from measurements for the patients from Nantes and Saint-Nazaire. Body mass index was obtained by dividing body mass by height squared.

During a first meeting at the structure, we interviewed each participant to determine their disability status using the Expanded Disability Status Scale (EDSS) [15]. The EDSS is a discrete scale that ranges from 0 to 10 points (with 0.5-point increments), with the highest scores being related to the worst disability status. In the present study, the EDSS was used to classify the participants as follows:

- ≤4 (can walk about 500 m or more without assistance or rest and can perform all their daily life activities);
- 4.5 (can walk about 300 m without assistance or rest and can perform almost all their daily life activities);
- 5.0 (can walk about 200 m without assistance or rest and cannot perform all their daily life activities);
- 5.5 (can walk about 100 m without assistance or rest and cannot work part-time without provisions);
- 6.0 (needs assistance [rest, aid, presence of another person] to walk about 100 m).

Following the interview, each participant completed the 6-min walking test. This test consisted of walking the longest distance as possible back and forth in a 30-m corridor with turnaround points at the extremities of the 30-m line. Instructions were given to the participant just before the test and standardized feedbacks were provided every minute to support the participant and to indicate the remaining time [16]. The total distance performed during the 6-min walking test is classically determined in the rehabilitation setting to reflect the patient’s ability to perform daily activities [16]. In the present study, the participants could use an assistive device to complete the test (14 participants used no assistance, and 4 participants used a cane or a crutch).

During a subsequent meeting at the structure, the participants had to complete a 40 to 60-min outdoor walking session in a place free of vehicles (Angers: 47°29′12.7″ N 0°33′29.0″ W; Nantes: 47°12′37.3″ N 1°30′06.7″ W; Saint-Nazaire: 47°16′02.4″ N 2°13′14.2″ W). Six to 7 days and 16 to 23 days separated the outdoor walking session from the 6-min walking test in 12 and 6 participants, respectively. The three walking places used in the study had small differences in terms of altitude and declivity (altitude change/travelled distance × 100) profiles. This can be observed using the interactive Supplemental Digital Content (SDC) 1 file that was built using corrected altitude data (rescaled to a minimum of 0) and GPS distance data recorded from three participants, with data interpolated every 20 m (please see the following subsection for detailed explanations regarding computation of corrected altitude and GPS distance). Of note, the main difference between the walking courses was that in Angers, the beginning and the end of the walking course was associated with greater declivity than in the other walking places. Except this point, the three walking courses were all related in containing little variation in declivity (i.e., between −2% and +2% depending on the considered 20-m section).

During the outdoor walking session, the participants had to walk, at usual pace, the longest possible distance without stopping. After a stop, the participants could resume walking after a free recovery duration to perform again the greatest possible walking distance without stopping, and so on up to when the a priori fixed time limit of the session was reached. Thus, the procedure implemented in the present study for the outdoor walking session was the same as that previously described for measuring outdoor walking capacity in patients with intermittent claudication [7,8,11], except that in the present study several participants could be tested during the same session. As during the 6-min walking test, the participants could use an assistive device to complete the test (12 participants used a Nordic pole, 3 participants used a cane or a crutch, and 3 used no assistance).

During the outdoor walking session, the participants were equipped with a DG100 GPS receiver (GlobalSat, Taipei, Taiwan) [14,17] that is composed of a unit (8.0 × 5.5 × 1.8 cm, weight ~60 g) and an antenna that was placed on the right shoulder of the participants. Six different DG100 units were used, with 2 to 5 outdoor walking sessions completed with each unit. The DG100 was set to record speed (Doppler method) and coordinates (latitude, longitude, altitude) at a 1-Hz sampling rate with the European Geostationary Navigation Overlay Service function enabled. The DG100 units were set and data were downloaded using a personal computer and manufacturer’s software (Data Logger Utility, version 1.1). The Data Logger Utility software was also used to export the GPS data to gpx. files for further analysis. The meteorological information at the time of the walking session was recorded using the Weather Underground website (https://www.wunderground.com, accessed on March–April 2019).

Both before the 6-min walking test and the GPS session, the participants completed the Fatigue Severity Scale [18] to provide information regarding their state of fatigue during the week where the tests were completed.

### 2.3. GPS Data Analysis

**Figure 1:**
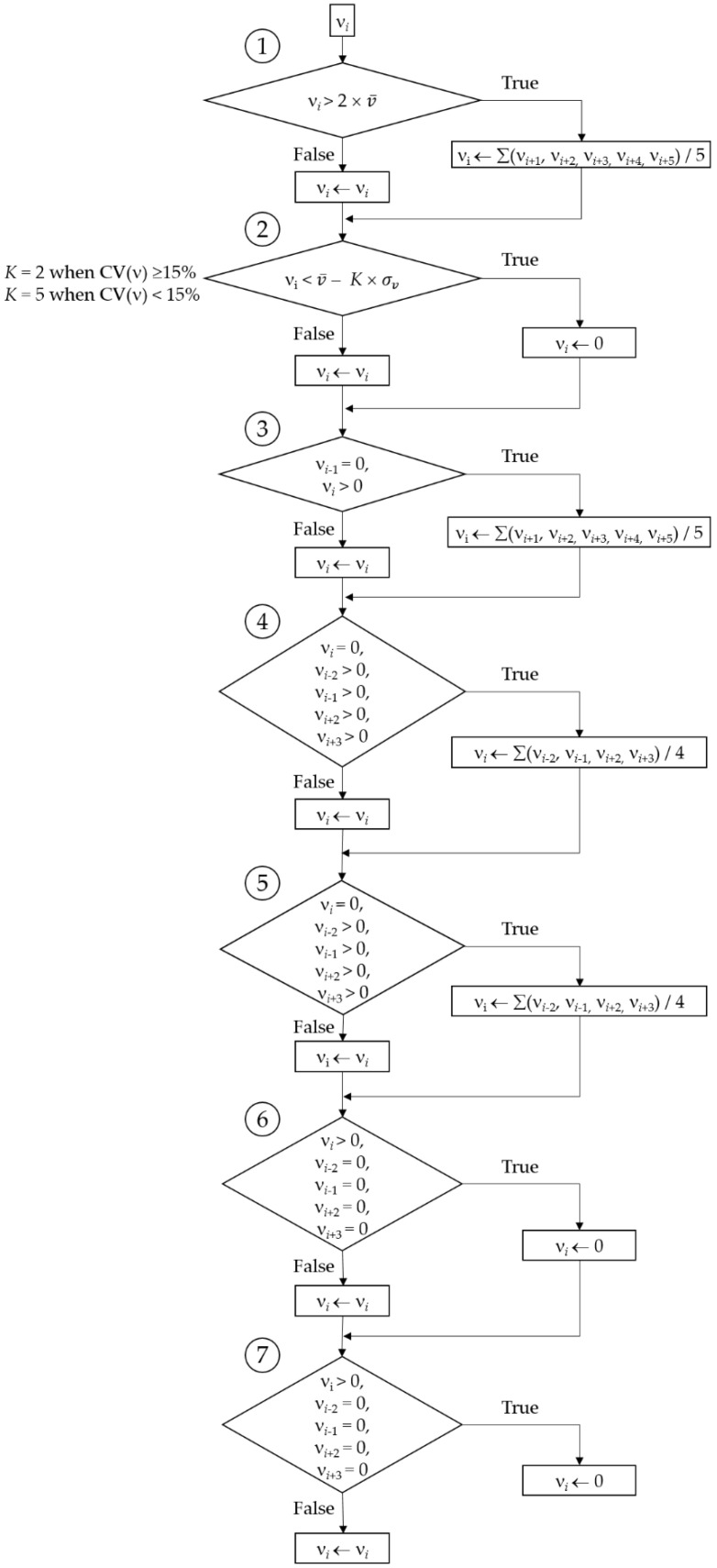
Block diagram illustrating the speed (ν) data processing methodology. $\bar{v}$, $\sigma_{v}$, CV(*ν*) are the mean, standard deviation, and coefficient of variation of speed over the representative 120-s (or shorter if not possible) period of the session; *νi* is the speed value corresponding to the epoch *i*. At each of the seven steps, *νi* outputs (*νi* ←) are new values that are included in a new vector *ν*.

**Figure 2:**
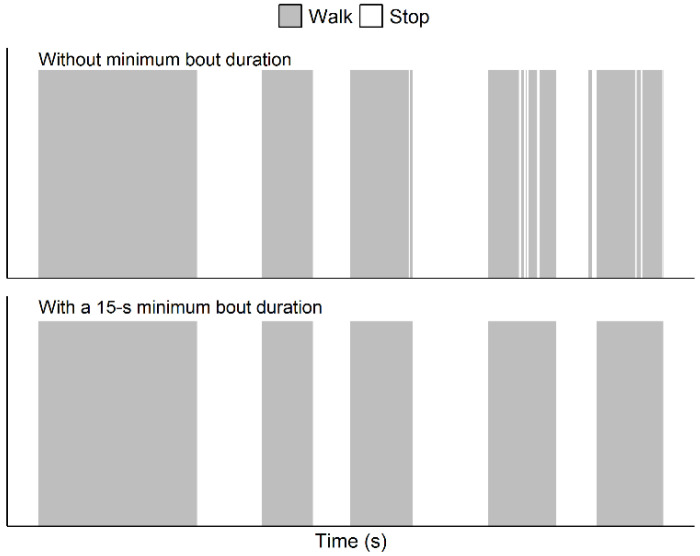
Illustration of the use of the 15-s minimum bout duration to detect the walking and stopping bouts during the evaluation of outdoor walking capacities using GPS.

We analyzed GPS data for participants who completed the outdoor session while respecting the provided rules. Analyses were performed using R software (version 3.6.0 (26 April 2019) [19]) and consisted of the followings steps (steps 2, 3 and 4 are based on previous works that studied the validity of a speed processing methodology for detecting walking and stopping bouts and estimating walking speed and distance [13,14]):

- Step 1, preparation of the dataset to have the required data in an appropriate format for further analysis (this step included the addition, in the dataset, of the altitude data from the French National Institute of Geography map projections obtained using CartoExploreur3 [version 3.11.0, build 2.8.10.14, Bayo, Ltd., Abrest, France] and that corresponded to the latitude and longitude data recorded by the GPS device; an illustration of the procedure to get these data is provided elsewhere [20]).
- Step 2, selection, based on graphical visualization, of the time period of interest (i.e., a few seconds before the first walking bout of the session to a few seconds after the last walking bout of the session); this step also allowed us to graphically confirm data validity (i.e., absence of spurious GPS signals that could prevent the possibility to detect the occurrence of a walking bout or a stopping bout).
- Step 3, calculations of the mean ($\bar{v}$), the standard deviation ($\sigma_{v}$), and the coefficient of variation (CV(ν)) of speed using a representative 120-s period (or less if not possible) to allow for the configuration of appropriate speed signal filters [13].
- Step 4, processing the speed data to remove noise and artifacts from the speed signal [13]. This step consisted of implementing several filtering and smoothing procedures (Figure 1). First, a filter was used to manage speed values that were greater than twice $\bar{v}$. Such values were replaced by the mean of the speed values corresponding to the five following epochs. Then, a second filter was used to set to 0 the speed values that were lower than a threshold value computed as follows: $\bar{v}$-*K* × $\sigma_{v}$; *K* being a coefficient set according to the value of CV(ν). When CV(ν) was ≥15%, *K* was set to 2, and when CV(ν) was <15%, *K* was set to 5. Then, a series of smoothing actions were sequentially performed on the speed data to remove very short walking and stopping bouts (i.e., bouts that corresponded to one or two 1-s epochs only). These actions were as follows:
  Action 1: For a given epoch *i*, if the speed value of the epoch *i* − 1 was 0 and the considered epoch *i* was greater than 0, then the value of the epoch *i* was shifted to the mean of the five following epochs.
  Action 2: For a given epoch *i*, if the speed value was 0 and the values of the epochs *i* − 2, *i* − 1, *i* + 2, and *i* + 3 were greater than 0, then the value of the epoch *i* was shifted to the mean of the speed values corresponding to the epochs *i* − 2, *i* − 1, i + 2, and *i* + 3.
  Action 3: Same action as Action 2.
  Action 4: For a given epoch *i*, if the speed value was greater than 0 and the values of the epochs *i* − 2, *i* − 1, *i* + 2, and *i* + 3 were 0, then the value of the epoch *i* was shifted to the mean of the speed values corresponding to the epochs *i*− 2, *i* − 1, *i* + 2, and *i* + 3, that is 0.
  Action 5: Same action as Action 4.
  Of note, this methodology actually managed shorter artifactual bouts than in the papers by Le Faucheur et al. [13] and Noury-Desvaux et al. [14]. Indeed, in the present study the methodology was implemented using epochs of a shorter duration (i.e., with epochs of 1 s due to a 1-Hz recording rate) than previously performed (i.e., with epochs of 2 s due to a 0.5-Hz recording rate [13]). In these previous studies [13,14], more than 90% of the walking and stopping bouts could be correctly detected using the speed processing methodology, with a typical error expressed as a coefficient of variation for estimating speed and distance <5% whatever the speeds and distances tested using the DG100 GPS receiver [14].
- Step 5, identification of the walking and stopping bouts based on speed information and a minimum bout duration of 15 s [13]. For this last step, the duration of a given bout was incremented using the durations of the subsequent detected bouts as long as that the bouts lasted less than 15 s, as illustrated in Figure 2. The minimum duration of 15 s to validate a shift from a walking bout to a stopping bout (and vice versa) was chosen because previous work [13] showed this was the shortest duration beyond which all bouts could be correctly detected using the present methodology, and this duration was thought to be sufficiently short to not correspond to a true and meaningful walking bout or stopping bout.

**Figure 3:**
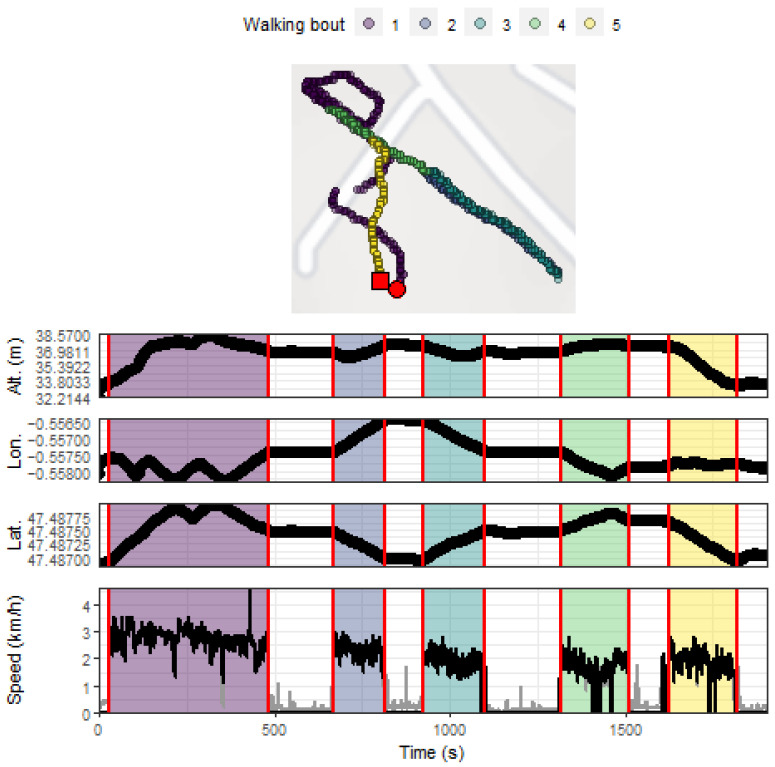
Example of GPS data obtained during an outdoor walking session (Participant #1). The upper panel depicts the positions recorded during the session, with the red circle and square indicating the beginning and the end of the session, respectively. The colored zones in the middle and lower panels highlight the walking bouts detected using the implemented speed processing methodology [13]. The altitude values are those from the French National Institute of Geography map projections that corresponded to the latitude and longitude data recorded by the GPS device. Grey lines depict raw speed values obtained with the GPS device and that were transformed following the speed data processing procedure. Lat. = latitude; Lon = longitude; Alt. = altitude.

After implementing this procedure, a visualization of the map combined with all GPS data (altitude, longitude, latitude, raw speed, processed speed, detected bouts) of the participant was produced. An example of the visualization obtained for one participant is shown in Figure 3.

From the processed GPS data file, we calculated, for each participant and where appropriate, the following outcomes: the session duration (from the beginning of the first walking bout to the end of the last walking bout), the total walking time, the total walking distance, the mean speed calculated using all the detected walking bouts, and the number of stops due to symptoms during the session. For the participants who stopped at least once during the session due to symptoms, we determined the greatest walking distance performed over a detected walking bout during the session (this parameter was considered as the session outdoor maximal walking distance in the present study), the mean and coefficient of variation (CV) of the performed walking distances, the mean and the CV of mean walking speed adopted during the walking bouts, the mean and the CV of stop durations, and the walking bout during which the greatest walking distance was recorded. Of note, for calculating these last outcomes, the last walking bout of the session was not included in the analysis when it did not correspond to the walking capacity, this to avoid the inclusion of a potential non-symptom limited walking bout, as previously performed [7].

### 2.4. Statistical Analysis

Statistical analysis was conducted using R software (version 3.6.0 (26 April 2019) [19]). Numeric variables are shown as median and interquartile range [IQR], and categorical variables are shown as counts and proportions throughout the article.

We calculated 90% confidence intervals (90% CI) for the medians and proportions related to the variables investigated during the outdoor walking session only. For the medians, the 90% CIs were calculated using the BCa method (999 replicates) implemented in the “groupwiseMedian” function of the “rcompanion” package [21]. For the proportions, the 90% CIs were calculated using the Clopper and Pearson method implemented in the “binom.test” function of the “stats” package [19].

We explored the construct validity of outdoor maximal walking distance (using the data from the participants who had to stop walking during the session) and outdoor usual walking speed (using the data from all the participants with valid results) as functional outcomes by investigating Pearson and Spearman correlations (and their respective 90% CIs) between those variables and the 6-min total walking distance.

### 2.5. Data and R Code Availability

All the participants’ GPS and corrected altitude data files, the R code that has been implemented to process these files, and the R code created for statistical analysis, are available in an Open Science Framework (OSF) repository (https://osf.io/tp37b, lastly updated on 17 April 2021). Of note, a personal Application Programming Interface (API) key is needed to get the map related to the GPS data when using the R code, but the proposed R functions allow getting the data visualizations without the map.

We also developed a shiny app available on the Web at https://pydemull.shinyapps.io/gps-walk to allow people who do not work with R to easily perform their GPS walking data analysis thanks to the same procedure as that used in the present article. In case the address at which the app is stored changes in the future, we provided the full code of the app in a Github repository (https://github.com/pydemull/gps-walk) in which the current valid address of the app is provided and will be updated when necessary.

## 3. Results

### 3.1. Participants Characteristics

Among the 18 participants initially included in the study, five were excluded due to GPS data loss (several data files were lost due to malfunction of one of the device used), and another participant was excluded as she did not respect the rules of the walking session (she was waiting for another participant and thus was not walking at her own pace). Thus, only 12 participants were included in final analysis. The characteristics of these 12 participants are shown in Table 1. Overall, participants were mainly women with normal weight with an EDSS score ≤ 4.5.

### 3.2. The Outdoor Walking Capacities

The outdoor walking sessions were completed with the following meteorological conditions: temperature of 13 [11–14] °C; relative humidity of 58 [55–66]%; wind speed of 19 [9–21] km/h. Among the 12 participants included in final analysis, 3 (25%) used no assistive device, 2 (17%) used a cane or a crutch, and 7 (58%) used Nordic poles. The FSS scores obtained at the group level at the time of the GPS session were similar to those obtained at the time of the 6-min walking test; however, some patients showed either a marked increase or a marked decrease in their FSS score between the 6-min walking test and the GPS evaluation periods (see SDC 2).

Most of the GPS data (>99%) were acquired with 1-s epochs for each participant, as expected. Only one participant (#9, see https://osf.io/rcjds for figure, lastly updated on 17 April 2021) had aberrant coordinates and speed data that corresponded to a stopping bout we detected using the speed processing methodology. It was deemed these spurious data were not a problem for getting reliable results since it seemed no displacement occurred during this period, as inferred from the coordinates that were the same at the beginning and at the end of this period.

Despite the initial target of completing a session of 40–60 min, eight participants stopped the session before reaching this target due to fatigue or personal time constraints. Thus, the median of the session time was only 30.4 min. The results regarding the variables measured during the outdoor walking session are shown in Table 2. While most of participants had a mean walking speed between 2 and 3 km/h, the slowest and fastest participants walked at 0.93 km/h and 3.84 km/h, respectively. Most of participants (10/12; 83%) had to stop walking at least once during the session.

Regarding the results of the participants who had ≥2 symptom-limited walking bouts, we could observe that walking distance during the different bouts and the stop durations were highly variable with a median CV > 50% and >70%, respectively. In contrast, mean walking speed was relatively stable over the different bouts with a median CV < 10% (Table 2).

**Figure 4:**
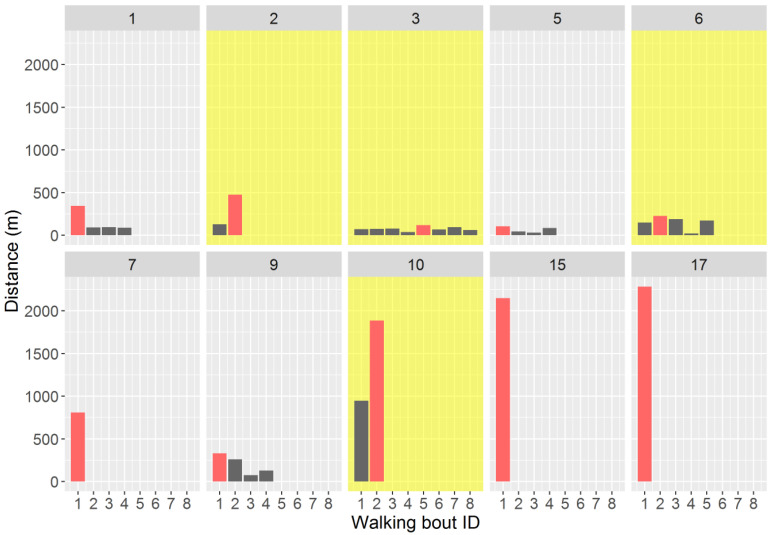
The symptom-limited walking distances performed during the walking bouts for each of the 10 participants who had to stop walking during the outdoor walking session. In a given panel, the red bar depicts the greatest distance performed over a walking bout during the session. The yellow panels highlight the participants who performed their greatest walking distance during the second or a subsequent walking bout.

Interestingly, among the 10 participants who stopped walking during the session, several (4/10; 40%) reached their greatest walking distance not during the first detected walking bout, but during one of the subsequent walking bouts. This result is highlighted in Figure 4.

### 3.3. Methodological Issues

**Figure 5:**
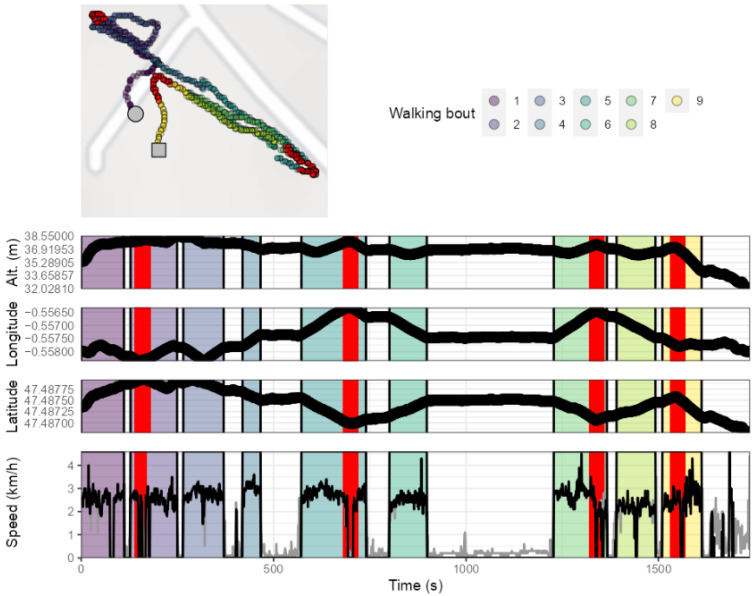
Illustrations of the methodological issues observed during GPS data analysis (here for Participant #3). The figure is one of the two examples without a clearly visible initial resting phase. The figure also shows that several parts of the proposed walking course (highlighted in red on the map), which were very steep turns, were related to a drop in walking speed. Grey lines depict raw speed values obtained with the GPS device and that were transformed following the speed data processing procedure. Alt. = altitude.

We encountered two methodological issues during data analysis. These issues are illustrated in Figure 5.

A first issue was that two participants’ (#2 and #3) data files did not show a resting phase at the beginning of the session. While the reason for this is not clear (e.g., device malfunction, loss of satellites, start of the session without all the required satellites), we are confident, based on the actual starting points, that the potential distance underestimation for the first walking bout of these two participants was within 20–30 m. This should have no effect on the greatest walking distance of the participants because this parameter was detected during one of the subsequent walking bouts with a much higher walking distance. However, this potential underestimation of walking distance during the first bout could influence the mean of the symptom-limited walking distances and the coefficient of variation of the symptom-limited walking distances performed during the session.

Graphical analysis also revealed that the walking courses we proposed challenged the ability of several participants to keep a constant walking speed during the session. Indeed, some parts of the walking courses could include very steep turns, which led to a drop in walking speed. This fact is illustrated for one participant in Figure 5.

### 3.4. Relationships Between Outdoor Walking Capacities and 6-min Total Walking Distance

**Figure 6:**
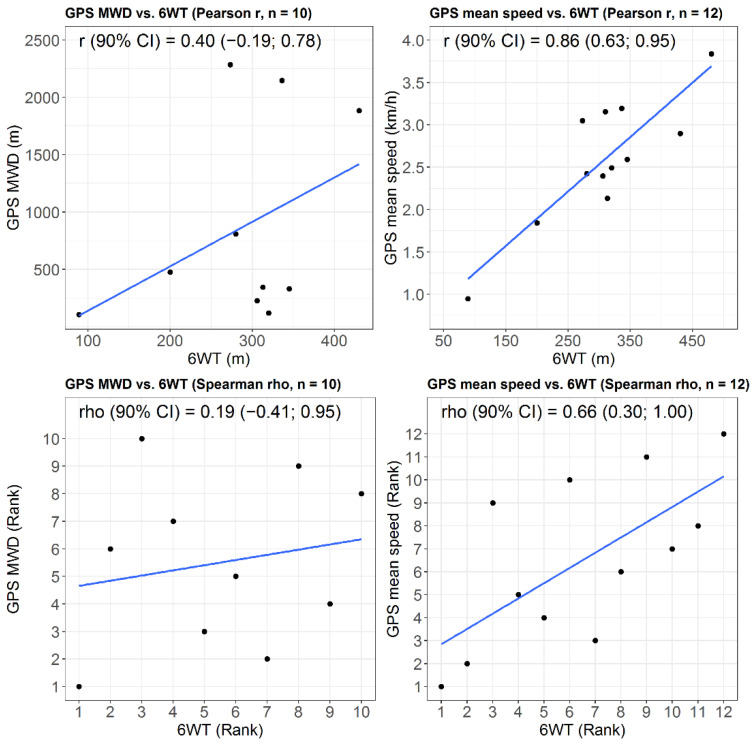
Relationships between outdoor walking capacities and the 6-min total walking distance. GPS MWD = Maximal walking distance measured using GPS during the outdoor walking session; 6 WT = Total walking distance measured during the 6-min walking test.

The results regarding the correlations between outdoor walking capacities and the 6-min total walking distance are shown in Figure 6. Pearson and Spearman correlations between outdoor maximal walking distance and 6-min total walking distance were low (≤0.40), with very wide 90% CIs that included 0. Pearson and Spearman correlations between outdoor mean walking speed and 6-min total walking distance seemed higher (≥0.65) with narrower 90% CIs.

## 4. Discussion

The present study aimed at exploring the GPS-measured outdoor walking capacities of people with MS assessed during a session that had to be performed at usual pace, with free recovery durations, and allowing multiple walking bouts. While this kind of evaluation has been implemented several times amongst people with intermittent claudication [7,8,11,22], this is, to our knowledge, the first results obtained in people with MS using such a procedure.

Unfortunately, it is difficult to compare our GPS-measured outdoor maximal walking distances with the two previous GPS studies conducted in people with MS [9,10]. Indeed, Dalla-Costa et al. [10] did not provide summary statistics for the recorded outdoor maximal walking distance. Moreover, while Créange et al. [9] obtained results from participants who had to walk until being forced to stop due to fatigue whatever the time needed to do this, we proposed in the present study an evaluation with an a priori fixed upper time limit. Thus, in our study, the least disabled participants did not reach a maximal walking distance during the session. This could explain why Créange et al. [9] obtained much higher values of outdoor maximal walking distance (median [IQR]: 1540 m [50–4550]) than in the present study.

Regarding outdoor usual walking speed, our participants could be considered as “slow walkers” in comparison with healthy people. Indeed, when considering all the walking time of the session, our participants walked at a median speed of 2.52 km/h (0.70 m/s), while a recent meta-analysis [23] estimated outdoor usual walking speed in healthy adults at a mean (95% CI) of 1.31 m/s (1.27; 1.35). The outdoor usual walking speed of our MS participants was also lower than that observed in other chronic disease populations, such as patients with intermittent claudication where a median [IQR] outdoor walking speed of 3.6 km/h [3.1–3.9 km/h] has been observed in a sample of 203 patients [8]. Our results, at the group level, are also lower than a recent estimate of the comfortable walking speed in people with MS [4], where authors found a mean speed (95% CI) of 1.12 m/s (1.05; 1.18). Thus, our sample of MS participants could be viewed as particularly disabled if we considered usual walking speed as a functional outcome [4]. Of note, because our methodology implemented a minimum bout duration of 15 s, the estimate of session mean walking speed could be underestimated because very short walking bouts that initially remained following speed data processing were related to low walking speeds. Including these bouts in ≥15-s bouts could have led to more weight being given to a walking bout with a lower mean speed in the final calculation of the session mean speed. To assess the impact of our methodology on the estimate of session mean speed, we conducted a sensitive analysis using no minimum bout duration to detect a given bout. This resulted in a median [IQR] (90% CI) walking speed of 2.64 km/h [2.22–3.05] (2.19; 2.93), that is, 0.12 km/h higher than following our initial analysis. Thus, the effect of the minimum bout duration of 15 s on session mean speed could be considered as not substantial because such variation corresponded to the typical error of estimate when speed is measured with the DG100 GPS during walking [14].

Interestingly, outdoor maximal walking distance was not always recorded during the first walking bout of the session. This was true for 40% of the participants among those who had to stop walking due to symptoms in the present study. While the reasons for which the walking distance performed between two stops could greatly vary from a waking bout to another are unclear, this result suggests that implementing an evaluation session allowing multiple walking bouts may be of interest to have a more reliable result of the outdoor maximal walking distance in people with MS. However, such a procedure could be worthwhile only in patients who present sufficient disability to have the possibility to perform several walking bouts during a session of a reasonable duration. Unfortunately, due to a small sample size, it was not possible here to reliably determine which disability status would be associated with the need to perform several walking bouts during a session, nor the session duration that would be needed to achieve the “true” maximal walking distance of the session. This last information could be worthwhile to avoid the implementation of an unnecessarily time and effort-consuming walking session.

Regarding the methodological issues we encountered, the absence of a resting phase in the GPS data at the beginning of the session for two participants was of importance since it could threaten the validity of the maximal walking distance recorded for those participants. This problem highlighted the need of graphical analysis when dealing with GPS data recorded in the present context, even if the R code we provided could work and provided results despite this loss of data. It could be good practice to determine a precise starting point of the walking session that could be recognized on a map, and to compare it, when necessary, with the recorded starting point to have confidence in the true beginning of the session observed in the GPS data. In the present study, we did not have such a precise starting point but we were able to situate the actual starting point with an approximated error of 20–30 m and ensure that the loss of data for the two concerned participants was not an issue in confirming their outdoor maximal walking distance.

The second methodological issue we met was that the walking courses we proposed could influence the walking speed in some participants. This fact could be observed in some of the figures created to show all the GPS data related to a given participant (e.g., Figure 5; see also all figures included in the OSF repository: https://osf.io/tp37b, lastly updated on 17 April 2021), with a walking speed that sometimes fell to nearly 0 km/h where turns were steep. The fall in walking speed in such cases could reveal a strategy of the concerned participants to maintain balance in these situations, as it has been observed and explained during 6-min walking tests with courses including 180° turns [24]. This issue may have influenced the walking distances performed over the walking bouts and necessarily led to a decrease in mean walking speed over the concerned walking bouts. Such falls in walking speed could have resulted in an increase in the number of detected bouts, but the minimal durations of 15 s we used to validate a bout seem to have prevented this. Thus, good practice for future implementations of GPS-based measurements of outdoor walking capacities in people with MS would be to design walking courses without steep turns to allow all participants to keep a relatively constant walking speed. This would allow easier analysis and easier comparisons of outdoor maximal walking distances and mean walking speeds between the participants in future studies.

Our results suggest that outdoor maximal walking distance might not well reflect walking endurance capacities as assessed using a 6-min walking test, with r and rho coefficients < 0.50, while mean outdoor walking speed could more likely discriminate against people with MS as it can be done based on the 6-min total walking distance (r and rho > 0.65). While our small sample of people with MS does not allow us to make accurate and definitive conclusions about the correlations between outdoor walking capacities and those assessed using the 6-min walking test, both outdoor maximal walking distance and walking speed may be interesting clinical information regarding how a patient is able to walk in natural contexts. Of note, a previous study [25] has shown that comfortable walking speed chosen during a maximal walking bout performed inside a clinic is a more stable parameter over different days in people with MS than maximal walking distance, meaning that walking speed would be more likely to allow the detection of change in disability status. This could also be the case for outdoor walking speed in comparison with outdoor maximal walking distance, but this should be investigated in reliability studies.

The present work presents several strengths. First, our results were obtained under normal working conditions related to three different healthcare or sports structures and highlight the possibility, and also the challenges, of conducting GPS-measurements in real-world contexts. Second, all of our GPS results are provided in a context that is transparently presented for all the participants, with the possibility to make judgements about the characteristics of the walking courses (i.e., based on corrected altitude data, longitude/latitude data, and the corresponding map) and about the quality of the GPS signals. Third, the present paper proposes fully open materials with de-identified information, allowing the reproduction of GPS data analysis and statistical analysis. Of note, if used for new investigations, the R code could need some adaptations depending on the structure of the gpx. files, and on the use or not of corrected altitude data.

Several limits have also to be acknowledged. First, during the outdoor walking session, some participants choose to use a walking aid although they were able to walk without it. Moreover, some participants could perform their walk relatively close to other participants. We do not know to what extent our GPS results may have been influenced by these elements, but this should be taken into account for potential comparisons with future studies. Second, our outdoor walking sessions had an upper time limit, forcing the participants to stop walking even if they could have walked a longer distance. Thus, our ability to detect and quantify outdoor maximal waking distance was restricted to the most disabled participants. Third, due to the fact that we used a convenience sample of small size and an exploratory approach, any estimate we provided should be confirmed and more robustly determined with appropriate confirmatory studies. Fourth, despite the fact that we sought to conduct the outdoor walking sessions in places where the walking courses were as flat as possible, we could observe some variations in the altitude profile of the walking courses depending on where the measurements were performed (see SDC 1). This might have differently influenced the walking capacities of the participants (i.e., outdoor maximal walking distance and mean walking speed). Future studies should prefer standardized environments (e.g., an athletic track) to assess outdoor walking capacities in people with MS, at least if the aim is to establish reference values and some relationships between outdoor walking capacities and other variables. However, clinicians could be interested in how patients adapt to variations in the physical environment (e.g., various grades, various terrain surfaces). In this case, evaluations could be performed throughout the day with no constraints about the features of the terrain to observe then how maximal walking distance or walking speed would vary depending on the difficulty of the terrain. Such evaluation would require further software development to put in relation distance, speed, and topography information, for example. Fifth, as we have used a recording rate (1 Hz) that was different from that used in previous validation works (0.5 Hz), it is difficult to precisely predict the accuracy with which we have detected the walking and stopping bouts when using the speed processing methodology.

To allow a more user-friendly experience than using R software when analyzing GPS walking data, we developed a shiny app available on the Web. Of note, a web platform just recently released (https://mapam.ens-rennes.fr, accessed on 2 April 2021) along with a published paper [26] now allows a full automatisation of speed data analysis, rather than manually building speed data filters that would be the best suited for the detection of walking and stopping bouts performed during the session. Unfortunately, the web platform does not yet provide contextual information (map, coordinates) nor a full summary of the walking session results as proposed when using the shiny app. Moreover, the overall workflow we used to get the final results may still be difficult to implement during clinical routines. This stresses the need of engineering work to develop software solutions, that would ideally be device-agnostic, personal computer-based, standalone and open source, to visualize GPS data and related context and to easily get the results of interest. Such software solutions could be combined with devices and applications allowing for patients’ tele-monitoring using GPS data to get the results in real-time. Some projects are already ongoing and conducted by other research teams to take up these kinds of methodological and technical challenges (https://project.inria.fr/sherpam, accessed on 2 April 2021).

From a clinical point of view, future studies could more precisely determine the proportion of people with MS that really need several walking bouts to reach their maximal walking distance, as well as investigate relationships between the well-established EDSS score and outdoor walking capacities. Moreover, it could be interesting to determine to what extent the ability to keep a constant pace over challenging outdoor walking courses (e.g., with 180° turns) could reflect the disability status of the patients; this could be valuable information when dealing with outdoor walking capacities in people with MS. Finally, future works could consider the assessment of gait quality during outdoor walking (e.g., using gait asymmetry or gait variability metrics) to better understand its potential effect on the quantity of walking that people with MS can perform (e.g., assessed using maximal walking distance as dependent variable).

## 5. Conclusions

The present study provides information regarding how maximal outdoor walking distance and walking speed could be assessed in people with MS during a session allowing multiple walking bouts to be performed, and also using a previously validated and described GPS speed processing methodology. We observed that the longest distance that a person with MS is able to walk before stopping due to their symptoms was not necessarily reached during their first walking bout during a session. Moreover, the outdoor maximal walking distances of people with MS did not seem related to the total distances performed during a standard 6-min walking test, while their mean outdoor walking speeds did. Further work is required to provide precise recommendations about the framework of the assessment of outdoor walking capacities in people with MS and to make easier the implementation of such assessments during clinical routine.

## Figures and Tables

**Table 1:** Participants Characteristics (*N* = 12).

Women, n (%)	11 (92)
Age, yr	55 [48–61]
Height, m	1.64 [1.59–1.67]
Body mass, kg	62 [57–64]
Body mass index, kg·m^−2^	21.75 [20.46–25.56]
EDSS score, n (%)	
≤4.0	4 (33)
4.5	4 (33)
5.0	1 (8)
6.0	3 (25)
6-min walking test	
FSS score the day of the test	5.5 [4.2–6.0]
Total walking distance, m	312 [278–338]
Device assistance	
No assistive device, n (%)	8 (67)
Cane or crutch, n (%)	4 (33)

Note Numeric variables are shown as median [IQR] and categorical variables as counts (proportions). FSS = Fatigue Severity Scale.

**Table 2:** GPS outdoor walking session.

All Participants (*N* = 12)	
FSS score	5.25 [4.38–5.85]
Session duration, min	30.4 [29.0–50.5] (28.8; 47.3)
Total walking time, min	23.6 [19.5–49.7] (19.5; 45.6)
Total walking distance, m	978 [709–2832] (710; 2190)
Mean speed over all the walking bouts, km/h	2.52 [2.19–3.04] (2.17; 2.93)
Participants with ≥1 stop (*N* = 10),%	83 (56; 97)
Participants with ≥1 stop (*N* = 10)	
Walking distance	
Max, m	410 [252–1615] (226; 1350)
Mean, m	251 [153–1263] (152; 1110)
CV,% (*N* = 7 with ≥2 walking bouts)	52.9 [49.1–69.7] (41.2; 58.9)
Walking speed	
Mean, km/h	2.46 [2.20–2.82] (2.13; 2.74)
CV,%	6.1 [5.2–14.4] (4.2; 10.9)
Stop duration	
Mean, min	2.5 [1.4–3.5] (1.3; 3.5)
CV,% (*N* = 5 with ≥2 stops)	70.4 [60.3–138.8] (34.4; 139.0)
Participants with the greatest walking distance recorded during the second bout or a subsequent one (*N* = 4),%	40 (15; 70)

Note Numeric variables are shown as median [IQR] and categorical variables as counts (proportions). Numbers in brackets are the 90% confidence intervals. FSS = Fatigue Severity Scale.

## Data Availability

The data presented in this study are openly available in Open Science Framework at 10.17605/OSF.IO/TP37B.

## Supplementary Materials

The following are available online at https://www.mdpi.com/article/10.3390/s21093189/s1, Figure S1: Supplemental Digital Content.

## References

[1] (2019). GBD 2016 Multiple Sclerosis Collaborators, Global, regional, and national burden of multiple sclerosis 1990–2016: A systematic analysis for the Global Burden of Disease Study 2016. Lancet Neurol..

[2] Brownlee W.J., Hardy T.A., Fazekas F., Miller D.H. (2017). Diagnosis of multiple sclerosis: Progress and challenges. Lancet.

[3] Sutliff M.H. (2010). Contribution of impaired mobility to patient burden in multiple sclerosis. Curr. Med. Res. Opin..

[4] Buoite Stella A., Morelli M.E., Giudici F., Sartori A., Manganotti P., di Prampero P.E. (2020). Comfortable walking speed and energy cost of locomotion in patients with multiple sclerosis. Eur. J. Appl. Physiol..

[5] Sandroff B.M., Klaren R.E., Motl R.W. (2015). Relationships among physical inactivity, deconditioning, and walking impairment in persons with multiple sclerosis. J. Neurol. Phys. Ther..

[6] Sparaco M., Lavorgna L., Conforti R., Tedeschi G., Bonavita S. (2018). The role of wearable devices in multiple sclerosis. Mult. Scler. Int..

[7] Le Faucheur A., Noury-Desvaux B., Mahe G., Sauvaget T., Saumet J.L., Leftheriotis G., Abraham P. (2010). Variability and short-term determinants of walking capacity in patients with intermittent claudication. J. Vasc. Surg..

[8] Gernigon M., Le Faucheur A., Noury-Desvaux B., Mahe G., Abraham P., Post-GPS Study Coinvestigators Group (2014). Applicability of global positioning system for the assessment of walking ability in patients with arterial claudication. J. Vasc. Surg..

[9] Créange A., Serre I., Levasseur M., Audry D., Nineb A., Boerio D., Moreau T., Maison P., Reseau S.-S. (2007). Walking capacities in multiple sclerosis measured by global positioning system odometer. Mult. Scler..

[10] Dalla-Costa G., Radaelli M., Maida S., Sangalli F., Colombo B., Moiola L., Comi G., Martinelli V. (2017). Smart watch, smarter EDSS: Improving disability assessment in multiple sclerosis clinical practice. J. Neurol. Sci..

[11] Le Faucheur A., Abraham P., Jaquinandi V., Bouye P., Saumet J.L., Noury-Desvaux B. (2008). Measurement of walking distance and speed in patients with peripheral arterial disease: A novel method using a global positioning system. Circulation.

[12] Gernigon M., Le Faucheur A., Fradin D., Noury-Desvaux B., Landron C., Mahe G., Abraham P. (2015). Global positioning system use in the community to evaluate improvements in walking after revascularization: A prospective multicenter study with 6-month follow-up in patients with peripheral arterial disease. Medicine.

[13] Le Faucheur A., Abraham P., Jaquinandi V., Bouye P., Saumet J.L., Noury-Desvaux B. (2007). Study of human outdoor walking with a low-cost GPS and simple spreadsheet analysis. Med. Sci. Sports Exerc..

[14] Noury-Desvaux B., Abraham P., Mahe G., Sauvaget T., Leftheriotis G., Le Faucheur A. (2011). The accuracy of a simple, low-cost GPS data logger/receiver to study outdoor human walking in view of health and clinical studies. PLoS ONE.

[15] Kurtzke J.F. (1983). Rating neurologic impairment in multiple sclerosis: An expanded disability status scale (EDSS). Neurology.

[16] American Thoracic Society (2002). ATS statement: Guidelines for the six-minute walk test. Am. J. Respir. Crit. Care Med..

[17] Abraham P., Noury-Desvaux B., Gernigon M., Mahe G., Sauvaget T., Leftheriotis G., Le Faucheur A. (2012). The inter- and intra-unit variability of a low-cost GPS data logger/receiver to study human outdoor walking in view of health and clinical studies. PLoS ONE.

[18] SFSEP Société Francophone de la Sclérose en Plaques. https://sfsep.org/echelle-fss/.

[19] R Core Team (2019). R: A Language and Environment for Statistical Computing.

[20] De Müllenheim P.Y., Chaudru S., Gernigon M., Mahé G., Bickert S., Prioux J., Noury-Desvaux B., Le Faucheur A. (2016). Accuracy of a low-cost global positioning system receiver for estimating grade during outdoor walking. Physiol. Meas..

[21] Mangiafico S.S. (2016). Summary and Analysis of Extension Program Evaluation in R, Version 1.18.1. https://rcompanion.org/.

[22] Nordanstig J., Broeren M., Hensater M., Perlander A., Osterberg K., Jivegard L. (2014). Six-minute walk test closely correlates to “real-life” outdoor walking capacity and quality of life in patients with intermittent claudication. J. Vasc. Surg..

[23] Murtagh E.M., Mair J.L., Aguiar E., Tudor-Locke C., Murphy M.H. (2021). Outdoor walking speeds of apparently healthy adults: A systematic review and meta-analysis. Sports Med..

[24] Cederberg K.L.J., Sikes E.M., Bartolucci A.A., Motl R.W. (2019). Walking endurance in multiple sclerosis: Meta-analysis of six-minute walk test performance. Gait. Posture..

[25] Albrecht H., Wotzel C., Erasmus L.P., Kleinpeter M., Konig N., Pollmann W. (2001). Day-to-day variability of maximum walking distance in MS patients can mislead to relevant changes in the Expanded Disability Status Scale (EDSS): Average walking speed is a more constant parameter. Mult. Scler. J..

[26] Taoum A., Chaudru S., de Müllenheim P.Y., Congnard F., Emily M., Noury-Desvaux B., Bickert S., Carrault G., Mahe G., Le Faucheur A. (2021). Comparison of activity monitors accuracy in assessing intermittent outdoor walking. Med. Sci. Sports Exerc..

