# Supplementary figures and images for: Measuring Outdoor Walking Capacities Using Global Positioning System in People with Multiple Sclerosis: Clinical and Methodological Insights from an Exploratory Study
Source: Sensors (Basel). 2021 May 4;21(9):3189. doi: 10.3390/s21093189 (PMC8125650; doi:10.3390/s21093189)

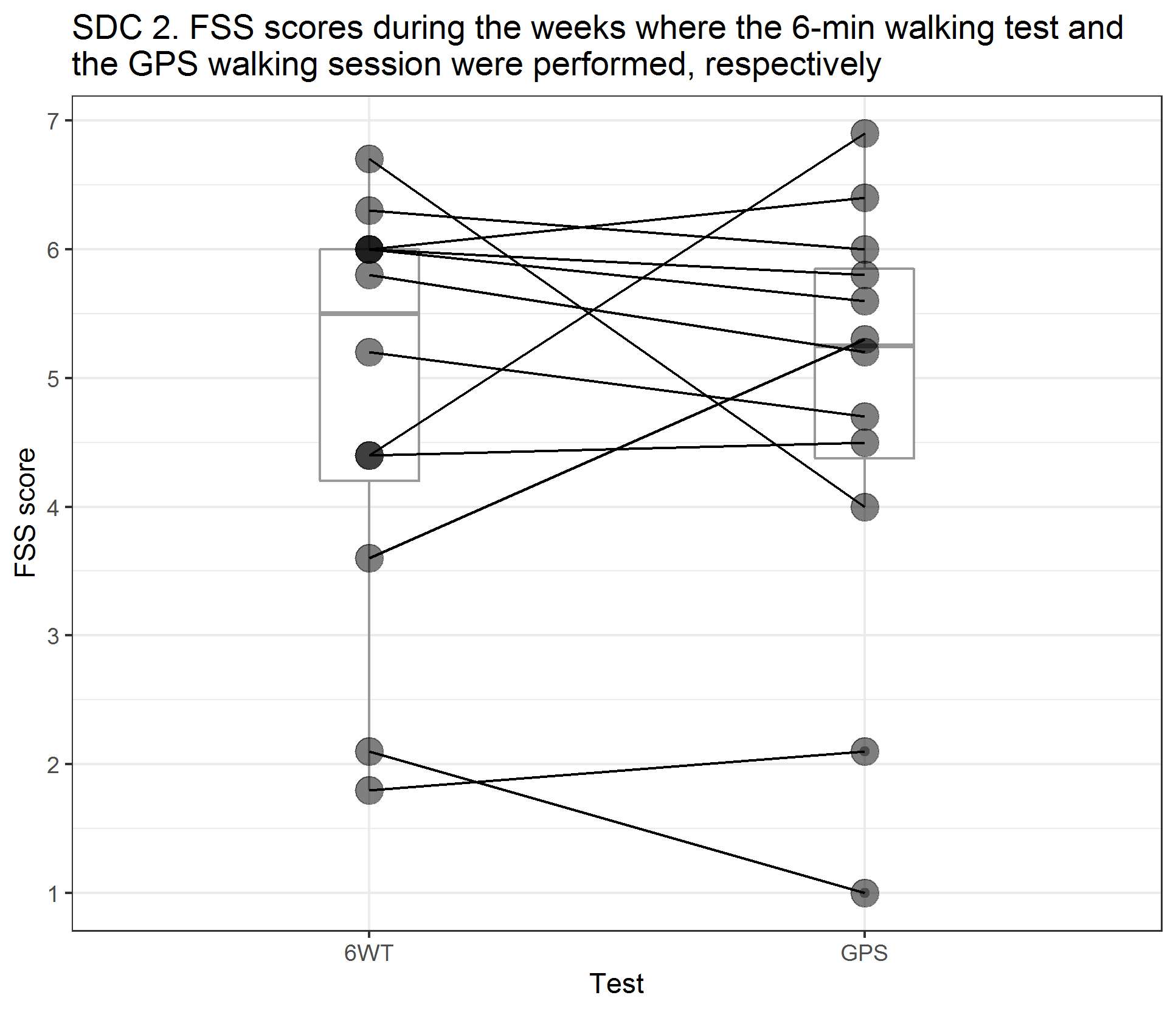

Supplement: Supplementary file 1 [file sensors-21-03189-s001.zip › SDC/SDC2.tiff]
